# Characterization of Cholinesterases in Plasma of Three Portuguese Native Bird Species: Application to Biomonitoring

**DOI:** 10.1371/journal.pone.0033975

**Published:** 2012-03-28

**Authors:** Cátia S. A. Santos, Marta S. Monteiro, Amadeu M. V. M. Soares, Susana Loureiro

**Affiliations:** Department of Biology and CESAM, University of Aveiro, Aveiro, Portugal; Weizmann Institute of Science, Israel

## Abstract

Over the last decades the inhibition of plasma cholinesterase (ChE) activity has been widely used as a biomarker to diagnose organophosphate and carbamate exposure. Plasma ChE activity is a useful and non-invasive method to monitor bird exposure to anticholinesterase compounds; nonetheless several studies had shown that the ChE form(s) present in avian plasma may vary greatly among species. In order to support further biomonitoring studies and provide reference data for wildlife risk-assessment, plasma cholinesterase of the northern gannet (*Morus bassanus*), the white stork (*Ciconia ciconia*) and the grey heron (*Ardea cinerea*) were characterized using three substrates (acetylthiocholine iodide, propionylthiocholine iodide, and S-butyrylthiocholine iodide) and three ChE inhibitors (eserine sulphate, BW284C51, and iso-OMPA). Additionally, the range of ChE activity that may be considered as basal levels for non-exposed individuals was determined. The results suggest that in the plasma of the three species studied the main cholinesterase form present is butyrylcholinesterase (BChE). Plasma BChE activity in non-exposed individuals was 0.48±0.11 SD U/ml, 0.39±0.12 SD U/ml, 0.15±0.04 SD U/ml in the northern gannet, white stork and grey heron, respectively. These results are crucial for the further use of plasma BChE activity in these bird species as a contamination bioindicator of anti-cholinesterase agents in both wetland and marine environments. Our findings also underscore the importance of plasma ChE characterization before its use as a biomarker in biomonitoring studies with birds.

## Introduction

Cholinesterase (ChE) activity has been routinely used as a biomarker to diagnose exposure to anticholinesterase compounds such as organophosphate (OP) and carbamate (CB) pesticides. These pesticides are broadly used to control insect pests and disease vectors; nonetheless, they can be extremely toxic to non-target organisms like mammals and birds [1], [2], [3], [4]. They act by inhibiting the activity of cholinesterases, which causes an over accumulation of acetylcholine at the synapses and consequent disruption of nerve function, leading to subsequent physiologic disorders and ultimately death [5]. In addition to OPs and CBs, other environmental contaminants such as metals, detergents and petroleum-derived products have been found to generate similar inhibitory effects [6], [7], [8]. Serum or plasma has been broadly used to measure ChE activity as a non-invasive method to monitor exposure of wildlife to pesticides in the field due to its sensitivity to ChE-inhibiting compounds [9], [10], [11], [12]. Nonetheless, its use requires the characterization of the enzyme form(s) present in the tissue assayed and the determination of the normal range of activity in non-exposed individuals [13].

Two enzymes form the family of cholinesterases: acetylcholinesterase (AChE; EC 3.1.1.7) and butyrylcholinesterase (BChE; EC 3.1.1.8). Both catalyze the hydrolysis of the neurotransmitter acetylcholine, but differ in substrate specificity and inhibitor susceptibility [14], [15]; tissue distribution can also vary, depending on the organism measured. AChE is predominantly found in the neuromuscular junctions and central nervous system, playing a key-role in the cholinergic neurotransmission, while BChE is mainly found in serum and liver, but its primary physiological role remains unknown [14].

Regarding wildlife exposure to environmental contaminants, waterbirds, such as wading birds and seabirds, are useful indicators of environmental variation upon short and long temporal scales [16]. Wading birds are primarily indicators of wetland quality as they can occupy a wide variety of foraging niches, including agricultural ponds, which makes them often non-target species to OP and CB exposure through ingestion and dermal contact [12]. In the case of seabirds, they are widely used to monitor the occurrence and ecological impacts of contaminants such as oil and mercury in the marine environment [17]. All these possible exposures may lead into an impairment of ChE activities in birds, and therefore biomarkers like this may be also a useful indicator to detect contamination in birds' habitats. In birds, AChE is found in the brain while BChE is mainly present in plasma; nonetheless, several studies had shown that AChE and other esterases (e.g. carboxylesterase- CbE) might also occur in avian plasma with wide interspecies differences [3], [18].

In order to use plasma ChE as a biomarker of exposure in three Portuguese native bird species, the main aim of this study was to: (i) characterize the ChE form(s) present in birds' plasma, (ii) determine the basal levels of ChE activity in non-exposed individuals and (iii) establish the appropriate assay conditions for the use of plasma ChE activity, using as bird species: the grey heron (*Ardea cinerea*) and the white stork (*Ciconia ciconia*), two wading bird species resident in Portugal, and the northern gannet (*Morus bassanus*), a migratory seabird common along the Portuguese coast during winter.

## Materials and Methods

### Sample collection

All the species of birds used to characterize plasma ChE were adult individuals inhabiting the Gaia Biological Park, a nature reserve located in Avintes (Porto, Portugal). *C. ciconia* individuals were free-living in the park while individuals of *A. cinerea* and *M. Bassanus* were in captivity. The disturbance stress caused by the animal handling was minimized by limiting the visit length, avoiding any sampling during extreme weather conditions (e.g. heavy rain, low temperatures) and using a small mantle to cover the head.

Blood was drawn from the brachial vein with sterile 1-ml syringes and 25-ga needles, and it was collected into a capillary tube with EDTA (Microvette® CB 300, Sarstedt). Following centrifugation, plasma was extracted and stored at −80°C until analysis.

### Sample preparation and ChE determinations

Plasma samples were diluted in phosphate buffer (0.1 M, pH 7.2), and ChE activity was determined in quadruplicate according to the Ellman method [19] adapted to microplate [20] using a microplate reader (Thermo Scientific Multiskan® Spectrum). For all species, plasma dilutions for each individual were prepared using 2 µl of plasma (2-µl micropipette, Gilson®) for a final assay volume of 1 ml. The enzymatic activity was expressed in units (U) per ml of plasma (1 U is a µmol of substrate hydrolyzed per minute).

### Cholinesterase characterization

Plasma ChE was characterized by testing the substrate preferences of the enzymes and their sensitivity to selective inhibitors. In independent experiments, acetylthiocholine iodide (AcSCh), S-butyrylthiocholine iodide (BuSCh) and propionylthiocholine iodide (PrSCh) were used as substrates at increasing concentrations (from 0.005 to 20.5 mM) and the enzymatic activity was determined.

Eserine sulphate, 1,5-bis(4-allyldimethyl-ammonimphenyl)pentan-3-one dibromide (BW284C51) and tetraisopropyl pyrophosphoramide (iso-OMPA) were selected as selective inhibitors of all ChE(s), AChE and BChE, respectively [21], [22]. For each inhibitor, stock solutions were prepared in ultrapure water or ethanol, as appropriate, with concentrations ranging from 6.25 to 200 µM (eserine and BW284C51) and from 0.25 to 8.0 mM (iso-OMPA). For each inhibitor, 5 µl of a stock solution was incubated with 495 µl of the sample during 30 min, at 25±1°C, before substrate addition. ChE was then assayed using both AcSCh and BuSCh as substrates. Ultrapure water was added to the controls and an additional control with ethanol was used in the experiments with iso-OMPA. In all characterization procedures, three samples of plasma corresponding to three adults per specie were used.

### Basal level for the activity of the dominant ChE

In order to determine the normal activity range of the dominant form of ChE present in the plasma of *M. bassanus*, *C. ciconia* and *A. cinerea*, samples of plasma from non-exposed individuals were assayed as previously described and the appropriated substrate was used. In this case, as BChE was the dominant form present (see details in the Results section) BuSCh was used as substrate at a concentration of 10.24 mM. The number of individuals ranged from 4–5 per bird species; sub-replicates of each individual ranged from 8–16.

### Chemicals

5,5′-Dithiobis(2-nitrobenzoic acid), AcSCh, BuSCh, PrSCh, Eserine hemisulphate, iso-OMPA, and BW284C51 were obtained from Sigma-Aldrich Europe (Netherlands). All the other chemicals used in this experiment were purchased from Merck (Germany).

### Data analysis

Analysis of variance (ANOVA) was performed to compare differences between inhibitor concentrations when the criteria of normality and equality of variance were satisfied (whenever necessary, data were transformed using log_10_ or square root). Dunnet's test was used to discriminate statistical differences between treatments and the control. All data analyses were performed using SigmaStat® 3.5 software (Systat Software Inc.).

### Ethics statement

All procedures involving bird handling were conducted according to the Guide for the Care and Use of Laboratory Animals of the European Union - in Portugal represented by Decreto de Lei n° 129/92 de 06 de Julho, Portaria n° 1005/92 de 23 de Outubro de 1992. Approval by a named review board institution or ethics committee was not necessary as the final model for ethical experimentation using animals is yet to be implemented in Portuguese research units.

## Results

**Figure 1:**
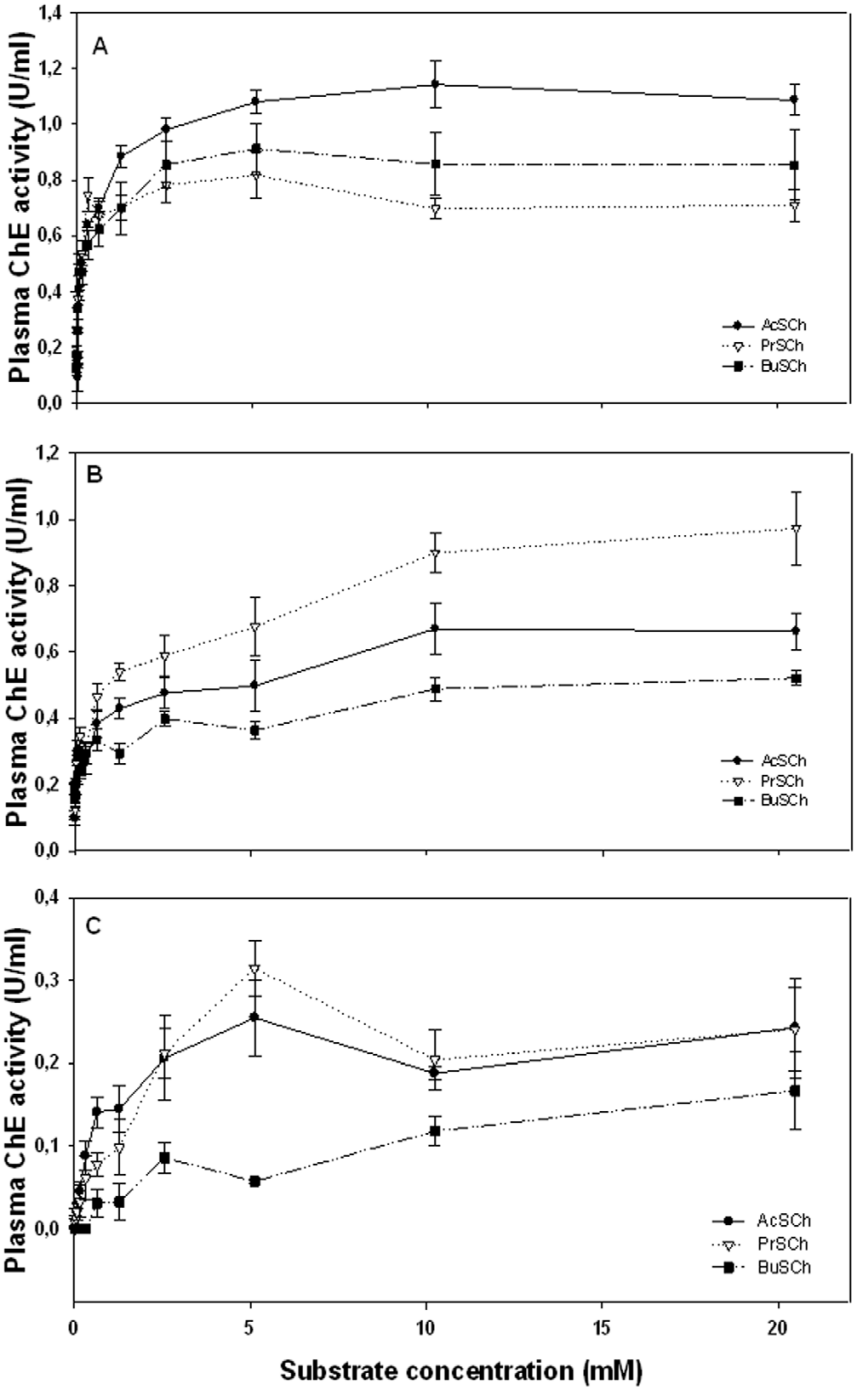
Plasma ChE activity at increasing concentrations of the substrates acetylthiocholine iodide (AcSCh), propionylthiocholine iodide (PrSCh) and *S*-butyrylthiocholine iodide (BuSCh) in: (A) *Morus bassanus*, (B) *Ciconia ciconia* and (C) *Ardea cinerea*. Results are expressed as the mean ± standard error of three birds.

In order to investigate substrate preferences of ChE in plasma of the species studied, the substrates AcSCh, PrSCh and BuSCh were assayed at increasing concentrations (Fig. 1). Maximum enzyme activity in *M. bassanus* was observed with AcSCh at 10.24 mM (1.14±0.08 SE U/ml), while in *C. ciconia* and *A. cinerea* maximum activity was obtained with PrSCh at 20.48 mM (0.97±0.11 SE U/ml) and 5.15 mM (0.32±0.03 SE U/ml), respectively. The enzyme kinetic parameters *V*_max_ (maximum rate of hydrolysis reached when the enzyme is saturated with substrate) and *K*_m_ (concentration needed to reach one-half of the maximum velocity) for the species studied are depicted in Table 1. Highest enzyme affinity in *M. bassanus* was obtained with AcSCh (K_m_ = 15.3 µM), while in *C. ciconia* was obtained with BuSCh (K_m_ = 4.8 µM) and *A. cinerea* with PrSCh (K_m_ = 150.1 µM).

**Table 1:** Apparent values of *K*_m_(µM) and *V*_max_(µmol/min/min) as estimated by Michaelis-Menten equation for the substrates AcSCh, BuSCh and PrSCh.

Species	AcSCh		BuSCh		PrSCh	
	K_m_	V_max_	K_m_	V_max_	K_m_	V_max_
*M. bassanus*	15.3	0.59	19.4	0.56	21.4	0.64
*C. ciconia*	10.7	0.27	4.8	0.29	6.1	0.34
*A. cinerea*	246.8	0.15	166.8	0.08	150.1	0.12

Values for this study are expressed as the mean value of 3 individuals.

Several works have indicated that most of the total ChE activity in plasma of different bird species is attributable to BChE [e.g. 23], [ 24]; therefore all inhibitor assays were performed using BuSCh, cleaved preferentially by BChE, and also using AcSCh, cleaved preferentially by AChE, both at the concentration of 10.24 mM. This allowed a better differentiation of the ChE form(s) present.

**Figure 2:**
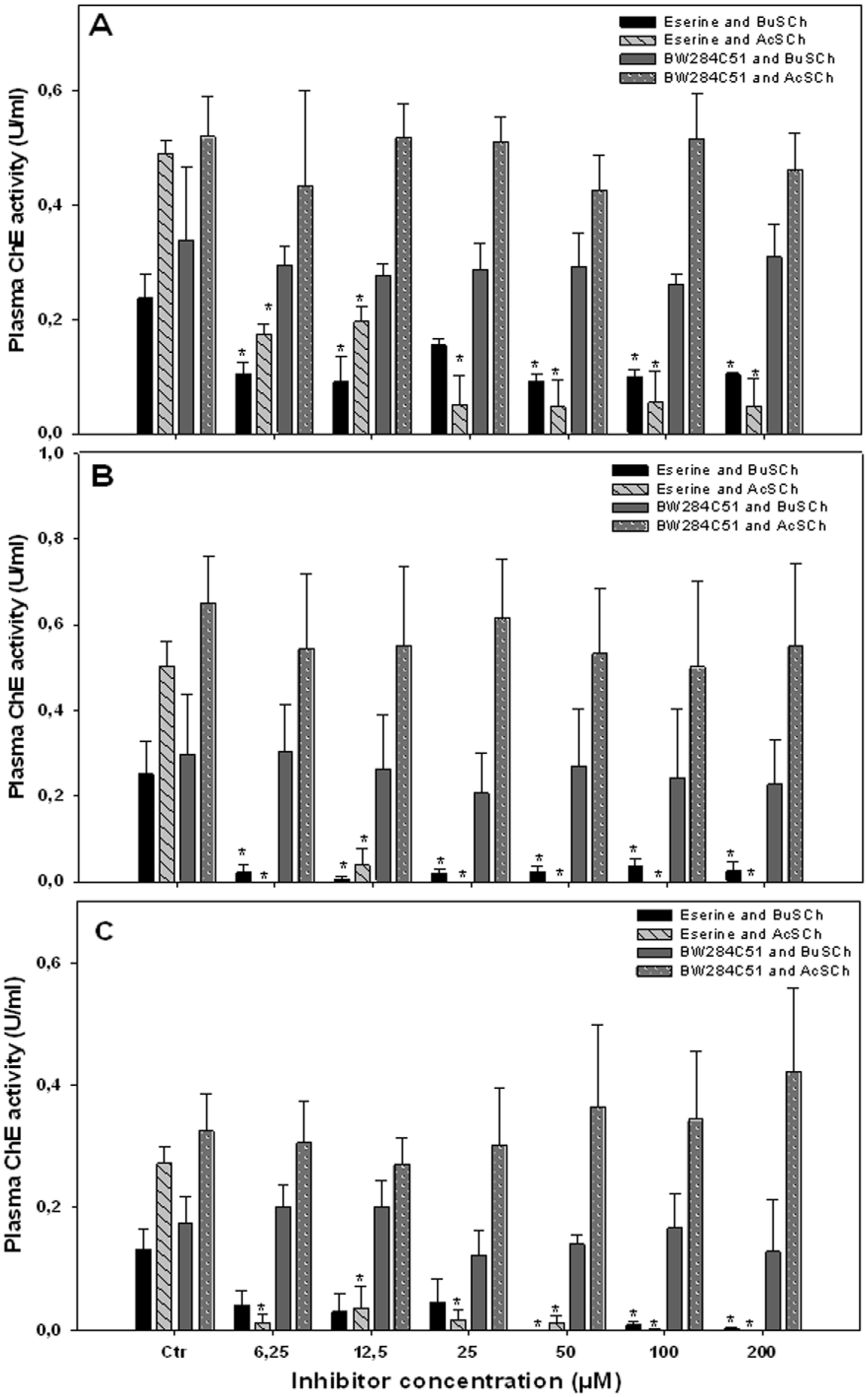
Effects of the inhibitors eserine and BW284C51 on the plasma ChE activity of: (A) *Morus bassanus*, (B) *Ciconia ciconia* and (C) *Ardea cinerea* using AcSCh or BuSCh as substrates. Results are expressed as the mean ± standard error of three birds; *significantly different from control (P<0.05) (Ctr =  control).

The effects of the inhibitor eserine sulphate are presented in Fig. 2. A clear inhibition of ChE activity (P<0.05) was observed in all species studied at the lowest inhibitor concentration using both substrates, except for *A. cinerea* (Fig. 2C) at 6.25–25 µM of eserine, when using BuSCh as a substrate. Inhibitions at 200 µM of eserine were about 56% and 91% in *M. bassanus*, 90% and 100% in *C. ciconia*, and 98% and 100% in *A. cinerea*, using BuSCh and AcSCh respectively. No effects on the ChE activity of the three species were reported when the selective inhibitor for AChE, BW284C51, was assayed at concentrations up to 200 µM (P<0.05), using both substrates (Fig. 2).

**Figure 3:**
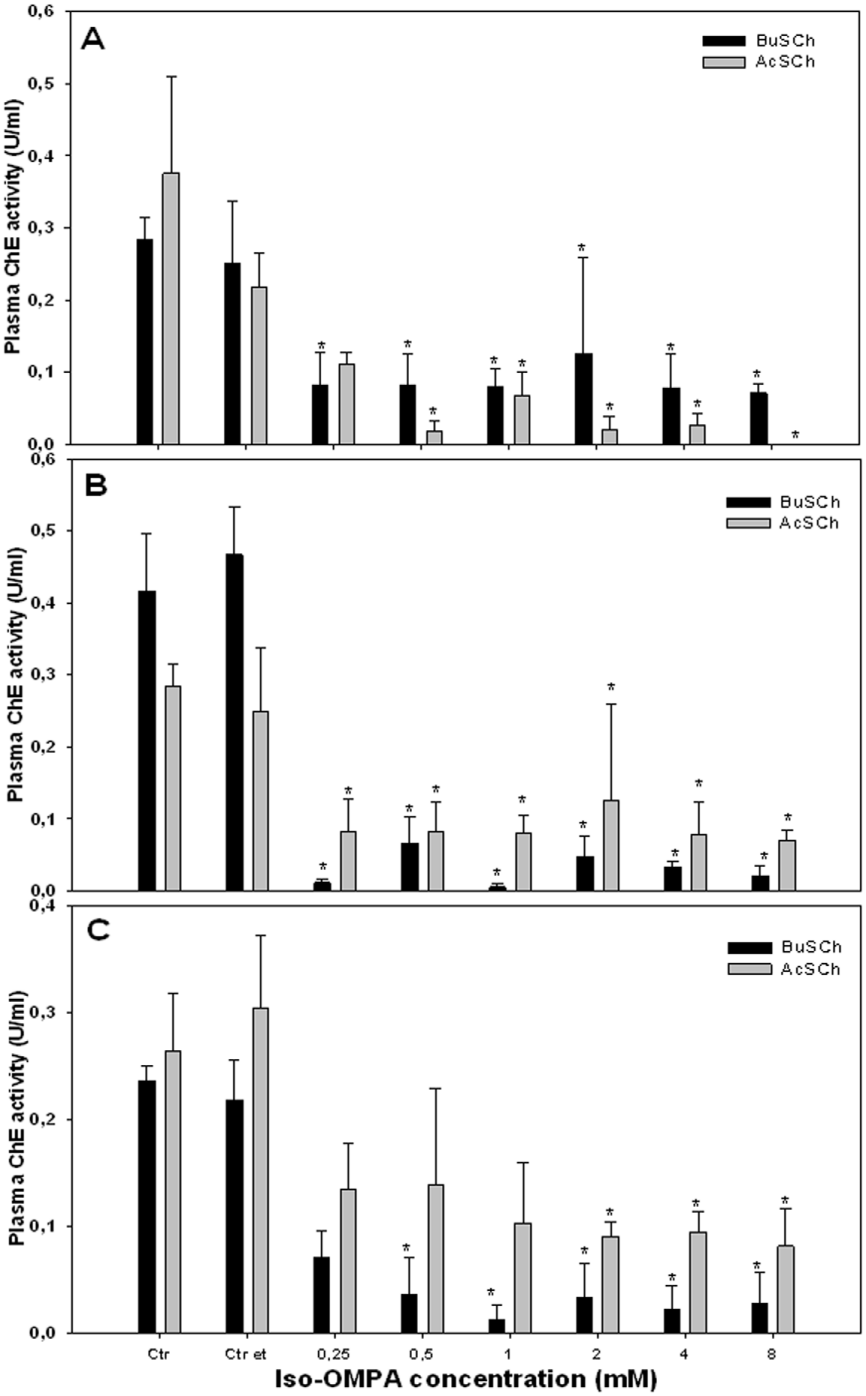
Effects of the inhibitor iso-OMPA on the plasma ChE activity of:(A) *Morus bassanus*, (B) *Ciconia ciconia* and (C) *Ardea cinerea* using AcSCh or BuSCh as substrates. Results are expressed as the mean ± standard error of three birds; *significantly different from control (*P<0.*05) (Ctr = control; Ctr et = control of the ethanol solvent).

In all species iso-OMPA, which is the selective inhibitor of BChE, significantly inhibited ChE activity in the presence of the substrates BuSCh and AcSCh (P<0.05) (Fig. 3). Inhibitions of about 82% with BuSCh and 100% with AcSCh in *M. bassanus* (Fig. 3A) were obtained at 8 mM of inhibitor. At the same concentration of iso-OMPA, inhibitions of about 95% and 97% were observed in *C. ciconia* (Fig. 3B), using BuSCh and AcSCh respectively. In *A. cinerea*, inhibitions of about 88% and 69% were observed with 8 mM of iso-OMPA, using BuSCh and AcSCh respectively.

The basal levels of BChE activity in plasma for the three species are depicted on Table 2. The variation in mean plasma BChE activity across species was 0.48±0.11 U/ml in *M. bassanus*, 0.39±0.12 U/ml in *C. ciconia* and 0.15±0.04 U/ml in *A. cinerea*.

**Table 2:** Normal range of ChE activity in non-exposed individuals, including sample size of individuals (n), minimum (min), maximum (max), mean and standard deviation (SD) values.

Species	BChE Activity (U/ml)				
	n	Min	Max	Mean	SD
*Morus bassanus*	5	0.36	0.64	0.48	0.107
*Ciconia ciconia*	5	0.26	0.54	0.39	0.124
*Ardea cinerea*	4	0.10	0.21	0.15	0.044

## Discussion

The first aim of this study was to characterize the ChE form(s) present in plasma of *M. bassanus*, *C. ciconia* and *A. cinerea*. In order to achieve that, the first step was to distinguish ChE form(s) from nonspecific esterases. This procedure is essential to avoid potential sources of error in biomarker studies because tissues may contain significant amounts of nonspecific esterases that might contribute to the total enzymatic activity measured, but differ in their sensitivities towards ChE-inhibiting compounds [13]. The presence of nonspecific esterases was determined using eserine sulphate, which is a selective inhibitor of all cholinesterase activity in the range of 10^−6^ to 10^−5^ M [21]. In this study, the enzymatic activity measured in plasma of the studied species was almost completely inhibited by eserine within the µM range, which indicates that the predominant enzyme(s) present is (are) ChE(s) and not other type of esterases.

In order to further distinguish which ChE form(s) was (were) present, the substrate preference of the enzyme and its sensitivity to BW284C51 and iso-OMPA was tested. ChE activity measured in plasma of *M. bassanus* showed a preference for AcSCh (Fig. 1A); in contrast, plasma ChE of *C. ciconia* and *A. cinerea* displayed a substrate preference towards PrSCh (Figs. 1B and 1C). Also, enzymatic activity was observed to stabilize at higher substrate concentrations, with no decrease of ChE activity. The higher rate of enzymatic activity registered with AcSCh in *M. bassanus* could be interpreted as a sign of the presence of AChE, although enzyme inhibition at higher substrate concentrations wasn't observed as it would be expected for a typical vertebrate AChE; additionally, a relatively high enzyme activity was obtained when using BuSCh (about 76% of the activity obtained with AcSCh at 5.12 mM), which is considered a specific substrate for BChE [23]. The other two bird species showed a preference towards PrSCh, but even though BuSCh was the substrate cleaved at the lowest rate, enzyme activity with this substrate was considerably high (about 54% and 78% of the activity obtained with PrSCh at 20.5 mM, in *C. ciconia* and *A. cinerea*, respectively). These results seem to indicate the presence of both ChE forms, AChE and BChE. This is in accordance with numerous studies which have indicated that most of the total ChE activity in plasma of birds is attributable to BChE, even though AChE activity may also occur at significant rates and the ratio between the two ChE forms may vary greatly among species [3], [18], [24]. After this first approach, to better characterize the ChE(s) present in plasma of the species studied, the inhibition assays with BW284C51 and iso-OMPA were performed using both AcSCh and BuSCh, in order to clarify which would be the most dominant form.

In all species studied, no effect on ChE activity was observed with BW284C51, selective inhibitor of AChE, using both AcSCh and BuSCh; furthermore, plasma ChE activity was strongly inhibited by iso-OMPA using both AcSCh and BuSCh. This seems to indicate the presence in all species of an enzyme that cleaves both AcSCh and BuSCh, that is resistant to BW284C51 (an AChE inhibitor) but highly sensitive to iso-OMPA (a specific inhibitor of BChE activity) [22]. Therefore, these results suggest that the enzyme present in plasma of the studied species is BChE. These results are in accordance with the majority of the studies published in this research area for birds. For example, Strum et al. [3] and Fildes et al. [24] have also found BChE to be the predominant ChE form present in the plasma of the sparrowhawk (*Accipiter nisus*), the Egyptian vulture (*Neophron percnopterus*), the brown songlark (*Cincloramphus cruralis*) and the australasian pipit (*Anthus novaeseelandiae*).

Like other vertebrates, in the present study plasma BChE activity of the northern gannet (*M. bassanus*), the white stork (*C. ciconia*) and the grey heron (*A. cinerea*) showed a Michaellis-Menten behaviour. Plasma BChE of *M. bassanus* and *C. ciconia* showed a higher affinity to BuSCh (K_m_ = 19.4 and 4.8 µM, respectively) than other vertebrates such as the mink *Mustela vison* (K_m_ = 240 µM) and the lizard *Gallotia galloti* (K_m_ = 1×10^3^ µM), but similar to the pigeon *Columbia livia* (K_m_ = 32 µM) and the rat *Rattus novergicus* (K_m_ = 13 µM) [25], [26]. The K_m_ values of the grey heron (*A. cinerea*) obtained with BuSCh was higher than the ones reported for the other two studied species, but smaller than the ones obtained with *M. vison*, *G. galloti* and the fish *Pioractus mesopotamicus* (K_m_ = 1.2×10^3^ µM) [25], [26].

Plasma BChE activity in non-exposed individuals of *M. bassanus*, *C. ciconia* and *A. cinerea* are within the range of values reported in the literature for several non-exposed birds. For example, mean BChE activity values measured for the kestrel (*Falco tinnunculus*), the buzzard (*Buteo buteo*) and the tawny owl (*Strix aluco*) were 0.28±0.08 SD U/ml, 0.91±0.8 SD U/ml and 1.49±0.36 SD U/ml, respectively [18]. Goldstein et al. [27] also measured a mean BChE activity of 0.41±0.09 SD U/ml in the swainson hawk (*Buteo swainsoni*), and Strum et al. [3] found BChE values of 0.83±0.01 SD U/ml in the American golden-plover (*Pluvialis dominica*) and 1.15±0.45 SD U/ml in the killdeer (*Charadrius vociferous*). The baseline plasma BChE values of the species presented in this study will provide a useful tool in the future for comparison with potential intoxicated/stressed birds but its use should be performed with caution. ChE activity may vary with several natural factors such as season, temperature and life stage [15]. Moreover, it should be taken into consideration that the sampling size of the obtained measurements is relatively low, even though a high number of replicates per individual were performed to decrease experimental error.

For future works with these species, it is recommendable the use of BuSCh at ∼10 mM, as it is a specific substrate for BChE and because in the species studied the maximum enzyme activity with this substrate was observed to be stable at that concentration.

In conclusion, the variability in the ChE activity of the species studied compared to other avian species previously studied underscores the importance of plasma ChE characterization before its use as a biomarker in biomonitorization studies with birds. For further use of plasma ChE activity as biomarker to diagnose exposure to anticholinesterase compounds in *M. bassanus*, *C. ciconia* and *A. cinerea*, BuSCh seems to be the most suitable substrate for enzymatic measurements, since BChE was found to be the predominant ChE form present in the species studied. The data presented here provide a starting point for the use of plasma BChE activity as a biomarker in Portuguese native bird species, aiding field investigations and monitoring risk of exposure of non-target wildlife to ChE-inhibiting compounds. In addition, the measurement of BChE activity may be a promising tool to be used in birds kept in captivity and recovering from physical or chemical stress, to evaluate their fitness and possible release.

## References

[1] Hill EF, Hoffman DJ, Rattner BA, Burton GA, Cairns J (2003). Wildlife Toxicology of Organophosphorus and Carbamate Pesticides.. Handbook of Ecotoxicology. Second ed.

[2] Blakley BR, Yole MJ (2002). Species differences in normal brain cholinesterase activities of animals and birds.. Veterinary and Human Toxicology.

[3] Strum KM, Alfaro M, Haase B, Hooper MJ, Johnson KA (2008). Plasma cholinesterases for monitoring pesticide exposure in Nearctic-Neotropical migratory shorebirds.. Ornitologia Neotropical.

[4] Grue CE, Gibert PL, Seeley ME (1997). Neurophysiological and behavioral changes in non-target wildlife exposed to organophosphate and carbamate pesticides: Thermoregulation, food consumption, and reproduction.. American Zoologist.

[5] Franson JC, Smith MR (1999). Poisoning of wild birds from exposure to anticholinesterase compounds and lead: Diagnostic methods and selected cases.. Seminars in Avian and Exotic Pet Medicine.

[6] Frasco MF, Fournier D, Carvalho F, Guilhermino L (2005). Do metals inhibit acetylcholinesterase (AChE)? Implementation of assay conditions for the use of AChE activity as a biomarker of metal toxicity.. Biomarkers.

[7] Guilhermino L, Barros P, Silva MC, Soares AMVM (1998). Should the use of inhibition of cholinesterases as a specific biomarker for organophosphate and carbamate pesticides be questioned?. Biomarkers.

[8] Soler F, Oropesa AL, Perez-Lopez M, Hernandez D, Garcia JP (2007). Acetylcholinesterase activity in seabirds affected by the Prestige oil spill on the Galician coast (NW Spain).. Science of The Total Environment.

[9] Cordi B, Fossi C, Depledge M (1997). Temporal biomarker responses in wild passerine birds exposed to pesticide spray drift.. Environmental Toxicology and Chemistry.

[10] Fossi MC, Massi A, Leonzio C (1994). Blood esterase inhibition in birds as an index of organophosphorus contamination: field and laboratory studies.. Ecotoxicology.

[11] McInnes PF, Andersen DE, Hoff DJ, Hooper MJ, Kinkel LL (1996). Monitoring exposure of nestling songbirds to agricultural application of an organophosphorus insecticide using cholinesterase activity.. Environmental Toxicology and Chemistry.

[12] Parsons KC, Matz AC, Hooper MJ, Pokras MA (2000). Monitoring Wading Bird Exposure to Agricultural Chemicals Using Serum Cholinesterase Activity.. Environmental Toxicology and Chemistry.

[13] Garcia LM, Castro B, Ribeiro R, Guilhermino L (2000). Characterization of cholinesterase from guppy (Poecilia reticulata) muscle and its in vitro inhibition by environmental contaminants.. Biomarkers.

[14] Radic Z, Taylor P, Gupta RC (2006). Structure and Function of Cholinesterases.. Toxicology of Organophosphate and Carbamate Pesticides.

[15] Monteiro M, Quintaneiro C, Morgado F, Soares AMVM, Guilhermino L (2005). Characterization of the cholinesterases present in head tissues of the estuarine fish Pomatoschistus microps: Application to biomonitoring.. Ecotoxicology and Environmental Safety.

[16] Amat JA, Green AJ, Hurford C, Schneider M, Cowx I (2010). Waterbirds as Bioindicators of Environmental Conditions.. Conservation Monitoring in Freshwater Habitats: A Practical Guide and Case Studies.

[17] Diamond AW, Devlin CM (2003). Seabirds as indicators of changes in marine ecosystems: Ecological monitoring on Machias Seal Island.. Environmental Monitoring and Assessment.

[18] Roy C, Grolleau G, Chamoulaud S, Rivere JL (2005). Plasma B-esterase activities in European raptors (vol 41, pg 184, 2005).. Journal of Wildlife Diseases.

[19] Ellman GL, Courtney KD, Andres V, Featherstone RM (1961). A New and Rapid Colorimetric Determination of Acetylcholinesterase Activity.. Biochemical Pharmacology.

[20] Guilhermino L, Celeste Lopes M, Carvalho AP, Soares AM (1996). Inhibition of acetylcholinesterase activity as effect criterion in acute tests with juvenile Daphnia magna.. Chemosphere.

[21] Eto M (1974). Organophosphorus Pesticides.

[22] Austin L, Berry WK (1953). Two selective inhibitors of cholinesterase.. Biochemical Journal.

[23] Sanchez-Hernandez JC, Plattenberg RH (2007). Ecotoxicological Perspectives of B-esterases in the Assessment of Pesticide Contamination.. Environmental Pollution: New Research.

[24] Fildes K, Szabo JK, Hooper MJ, Buttemer WA, Astheimer LB (2009). Plasma cholinesterase characteristics in native Australian birds: significance for monitoring avian species for pesticide exposure.. Emu.

[25] Moralev SN, Rozengart EV, Moralev SN, Rozengart EV (2007). Specificity of Cholinesterases in Reaction with Substrates.. Comparative enzymology of cholinesterases.

[26] Sanchez-Hernandez JC, Sanchez BM (2002). Lizard cholinesterases as biomarkers of pesticide exposure: Enzymological characterization.. Environmental Toxicology and Chemistry.

[27] Goldstein MI, Lacher TE, Zaccagnini ME, Parker ML, Hooper MJ (1999). Monitoring and Assessment of Swainson's Hawks in Argentina Following Restrictions on Monocrotophos Use, 1996–97.. Ecotoxicology.

